# Selection of Biomarkers and Clinical Endpoints for Clinical Trials Targeting Down syndrome‐related Alzheimer’s disease (DSAD)

**DOI:** 10.1002/alz.092032

**Published:** 2025-01-09

**Authors:** Michael Rafii

**Affiliations:** ^1^ Alzheimer's Therapeutic Research Institute, San Diego, CA USA

## Abstract

**Background:**

Major advances have been made over the past decade in understanding Down syndrome‐related AD by utilizing the latest AD biomarkers such as brain imaging and biofluid assays. Cognitive measures that can discern decline related to AD have been developed and validated in people with DS. We are now able to incorporate these data to help design clinical trials against AD in people with DS.

**Method:**

The NIH‐funded Alzheimer’s Clinical Trial Consortium ‐ Down Syndrome (ACTC‐DS) was launched in 2018 to serve as a platform for conducting clinical trials to treat and prevent AD dementia in people with DS. We have enrolled over 200 adults with DS who will be offered the opportunity to enroll in interventional clinical trials for AD specifically designed for people with DS.

**Result:**

We have launched a phase 1b/2, multicenter, double‐blind, randomized, placebo‐controlled study to assess the safety, tolerability, immunogenicity, and pharmacodynamic effects of ACI‐24.060 in in adults with Down syndrome and plan to launch two additional trials this year.

**Conclusion:**

Data from longitudinal biomarker studies are informing the design and conduct of clinical trials targeting AD in people with DS.

